# Chikusetsu Saponin V Attenuates MPP^+^-Induced Neurotoxicity in SH-SY5Y Cells via Regulation of Sirt1/Mn-SOD and GRP78/Caspase-12 Pathways

**DOI:** 10.3390/ijms150813209

**Published:** 2014-07-28

**Authors:** Ding Yuan, Jing-Zhi Wan, Li-Li Deng, Chang-Cheng Zhang, Yao-Yan Dun, Yan-Wen Dai, Zhi-Yong Zhou, Chao-Qi Liu, Ting Wang

**Affiliations:** 1 Department of Pharmacology, College of Medical Science, China Three Gorges University, Yichang 443002, China; E-Mails: yxyydyd@126.com (D.Y.); 15071798496@163.com (J.-Z.W.); denglili3748@163.com (L.-L.D.); dyydxy2004@163.com (Y.-Y.D.); dyw891122@163.com (Y.-W.D.); in_bud@ctgu.edu.cn (Z.-Y.Z.); 2 Third-Grade Pharmacological Laboratory on Chinese Medicine Approved by State Administration of Traditional Chinese Medicine, China Three Gorges University, Yichang 443002, China; 3 Institute of Molecular Biology, China Three Gorges University, Yichang 443002, China; E-Mail: ctgulcq@163.com

**Keywords:** Chikusetsu saponin V, MPP^+^, Sirt1, GRP78

## Abstract

Studies have shown that saponins from *Panax japonicus* (SPJ) possess neuroprotective effects. However, whether Chikusetsu saponin V (CsV), the most abundant member of SPJ, can exert neuroprotective effects against 1-methyl-4-phenylpyridinium ion (MPP^+^)-induced cytotoxicity is not known. In this study, we aimed to investigate the neuroprotective effects of CsV on MPP^+^-induced cytotoxicity in human neuroblastoma SH-SY5Y cells and explore its possible mechanisms. Our results show that CsV attenuates MPP^+^-induced cytotoxicity, inhibits ROS accumulation, and increases mitochondrial membrane potential dose-dependently. We also found that levels of Sirt1 protein and Mn-SOD mRNA significantly decreased in MPP^+^-treated group but were restored with CsV treatment in a dose-dependent manner. Furthermore, GRP78 protein and Caspase-12 mRNA levels were elevated by MPP^+^ exposure but reversed by CsV treatment. CsV inhibited the MPP^+^-induced downregulation of Bcl-2 and up-regulation of Bax in a dose-dependent manner and, thus, increased the ratio of Bcl-2/Bax. Overall, these results suggest that Sirt1/Mn-SOD and GRP78/Caspase-12 pathways might be involved in the CsV-mediated neuroprotective effects.

## 1. Introduction

Parkinson disease (PD) is a neurodegenerative disorder characterized by progressive degeneration of dopaminergic neurons in the substantia nigra pars compacta and decreased levels of dopamine in the putamen of dorsolateral striatum [1]. 1-Methy-4-phenyl-1,2,3,6-tetrahydropyridine (MPTP) and its active metabolite, 1-methyl-4-phenylpyridinium ion (MPP^+^), have been widely used to establish PD models *in vivo* or *in vitro* [2]. Evidence has suggested that MPP^+^ disrupts mitochondrial respiratory function via inhibiting mitochondrial complex I, which leads to impaired mitochondrial energy metabolism and oxidative stress [3]. The resulting oxidative stress and defective mitochondrial energy metabolism may initiate a cascade of biochemical and pathological events that lead to the widespread neuronal degeneration that resembles PD [4].

Although the pathological features of PD have been well described, the etiology remains undefined. Many studies have indicated that oxidative stress is involved in the pathogenesis of PD [4,5]. Sirt1, an NAD^+^-dependent class III histone deacetylase, is an important regulator of cell survival and lifespan [6]. A growing body of evidence suggests that Sirt1 increases cell survival and resistance to oxidative stress through several pathways [7,8]. Moderate over-expression of Sirt1 protects the heart from oxidative stress through up-regulation of antioxidants, such as Mn-SOD [9]. Oxidative stress, resveratrol or tetrahydroxystilbene glucoside treatment has been shown to activate Sirt1 [7,10]. All of the above suggest that Sirt1 could possibly promote neuronal survival against oxidative stress [11] through the Sirt1/Mn-SOD pathway.

Meanwhile, increasing evidence demonstrates that endoplasmic reticulum (ER) stress, together with abnormal protein degradation, contributes to the pathophysiology of PD [12,13]. ER stress activates the unfolded protein response that induces ER-associated protein degradation as a self-protective mechanism, thereby leading to rescue or adaptive responses [14]. Consequently, the unfolded protein response that initially aims to restore homeostasis could also mediate apoptosis if the stress cannot be removed [15]. It is currently understood that glucose regulated protein-78 (GRP78) serves as the monitor to detect accumulation of misfolded/unfolded proteins and plays a crucial role in the regulation of the ER dynamic homeostasis [16]. It has been reported that ER stress stimulus-induced cell apoptosis is mediated by the activation of Caspase-12 [17]. In addition to activation of ER-associated Caspase-12, C/EBP homologous protein (CHOP), a transcription factor that sensitizes the cells to apoptosis, is usually activated during ER stress [18].

**Figure 1:**
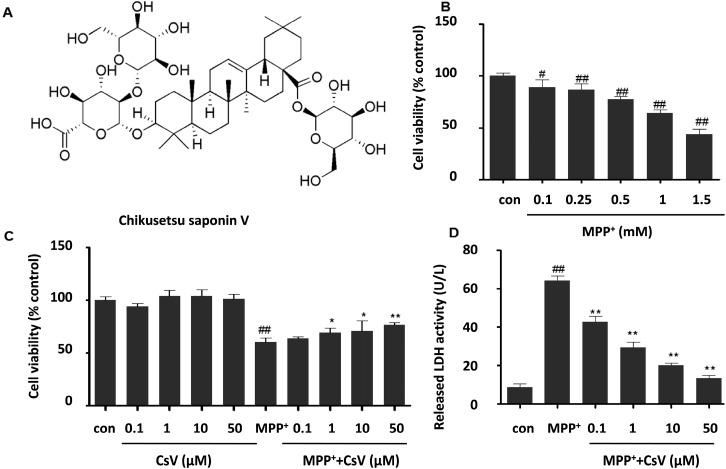
Protective effects of Chikusetsu saponin V (CsV) on 1-methyl-4-phenylpyridinium ion (MPP^+^)-induced cytotoxicity in SH-SY5Y cells. (**A**) Chemical structure of CsV; (**B**) Dose-dependent effects of MPP^+^ on cell viability in SH-SY5Y cells; (**C**) Effects of CsV on MPP^+^-induced cytotoxicity in SH-SY5Y cells as assessed by MTT assay; and (**D**) Effects of CsV on MPP^+^-induced cytotoxicity in SH-SY5Y cells as shown by Lactate dehydrogenase (LDH) assay. Data are expressed as means ± SD (*n* = 5). ^#^ *p* < 0.05, ^##^ *p* < 0.01 *vs.* control group, * *p* < 0.05, ** *p* < 0.01 *vs.* the group treated with MPP^+^ alone.

*Rhizoma Panacis japonica,* the dried rhizome of *Panax japonicus,* is a common traditional herbal medicine in Tujia and the Hmong people of China. It has been used as substitute for Ginseng roots [19,20]. Saponins from *Panax japonicus* (SPJ) are believed to be the most abundant and bioactive members in *Panacis japonica rhizoma* with anti-oxidative, anti-inflammatory, immunoregulatory, hepatoprotective, and neuroprotective effects [20,21,22]. SPJ includes Chikusetsu saponin I, IV, V, pjs1, pjs2, pjs4, *etc.* [23]. However, whether Chikusetsu saponin V (CsV, Chengdu Purechem-Standard Co., Ltd., Chengdu, China, Figure 1A), an abundant member of SPJ, can exert neuroprotective effects remains unknown [20].

SH-SY5Y cells are human neuroblastoma cells, which possess many characteristics of dopaminergic neurons and have been widely used in the study of cell model for PD [24]. Therefore, the goal of this study was to investigate the neuroprotective effects of CsV on MPP^+^-induced cytotoxicity in SH-SY5Y cells and potential mechanisms.

## 2. Results

### 2.1. Chikusetsu Saponin V (CsV) Attenuated 1-Methyl-4-phenylpyridinium Ion (MPP^+^)-Induced Cytotoxicity in SH-SY5Y Cells

In order to evaluate the cytotoxicity of CsV or MPP^+^, SH-SY5Y cells were incubated with different concentrations of CsV (0.1, 1, 10, 50 μM) or MPP^+^ (0.1, 0.25, 0.5, 1, 1.5 mM) for 24 h, and the cell viability was determined by MTT. Our results show a dose-dependent decrease in cell viability following MPP^+^ exposure (Figure 1B). The cell viability decreased to 64.3% ± 3.0% compared to the control group after 24 h exposure to 1 mM MPP^+^. Thus, this protocol was selected for further experiments in the present study.

To investigate the effects of CsV on MPP^+^-induced cell viability, cells were incubated with different concentrations of CsV for 24 h while exposed to 1 mM MPP^+^. As shown in Figure 1C, cell viability exposed to 1 mM MPP^+^ for 24 h was reduced to 60.3% ± 3.8% compared to the control (*p* < 0.01). However, treatment with CsV (0.1, 1, 10 and 50 µM) significantly attenuated MPP^+^ induced loss of cell viability in a dose-dependent manner and the cell viability significantly increased to 63.9% ± 1.2%, 68.9% ± 4.6%, 70.7% ± 9.9%, and 76.8% ± 2.0%, respectively. Meanwhile, no cytotoxic effect of CsV was observed in our experiments. The cytoprotective effects of CsV were also confirmed by Lactate dehydrogenase (LDH) assay. MPP^+^ treatment induced about 7.5-fold higher of LDH release compared to the control group, which was alleviated by CsV treatment dose dependently (Figure 1D).

### 2.2. CsV Suppressed MPP^+^-Induced ROS Accumulation in SH-SY5Y Cells

**Figure 2:**
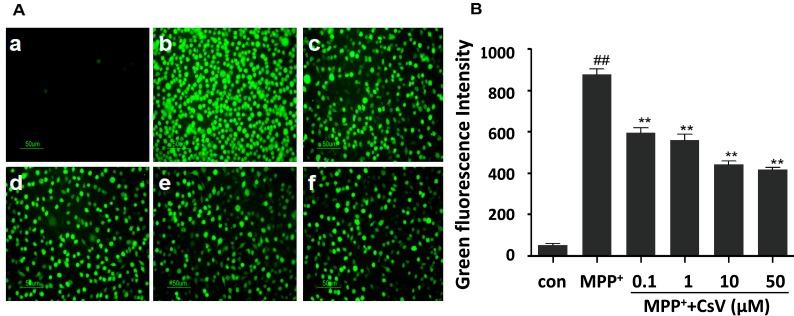
CsV suppressed MPP^+^-induced ROS accumulation in SH-SY5Y cells. (**A**) Cells were observed and photographed under a fluorescence microscope. Green fluorescence represented the amounts of ROS. Images shown are representative of five independent experiments. 100× magnification. (**a**) Control (Cells without MPP^+^ and CsV); (**b**) 1 mM MPP^+^ treatment alone; (**c**) 0.1 µM CsV with 1 mM MPP^+^; (**d**) 1 µM CsV with 1 mM MPP^+^; (**e**) 10 µM CsV with 1 mM MPP^+^; and (**f**) 50 µM CsV with 1 mM MPP^+^; and (**B**) Green fluorescence intensity was analyzed with the Image J (Image J Software, Materialize NV, Leuven, Belgium). ^##^ *p* < 0.01 *vs.* control group, ** *p* < 0.01 *vs.* MPP^+^ alone. Data are expressed as means ± SD (*n* = 5).

To examine whether the protective effect of CsV against MPP^+^-induced cytotoxicity was mediated by its antioxidant ability, the intracellular ROS levels were measured in SH-SY5Y cells. 2,7-dichlorfluorescein diacetate (DCFH-DA) was used as a fluorescence probe to assess intracellular ROS concentration. As shown in Figure 2, exposure of SH-SY5Y cells to 1 mM MPP^+^ for 24 h led to significant increase in DCF signal compared to the control group. Such increase in DCF fluorescence was reversed by treatment with CsV in a dose-dependent manner.

### 2.3. CsV Restored Mitochondrial Membrane Potential of SH-SY5Y Cells Treated with MPP^+^

**Figure 3:**
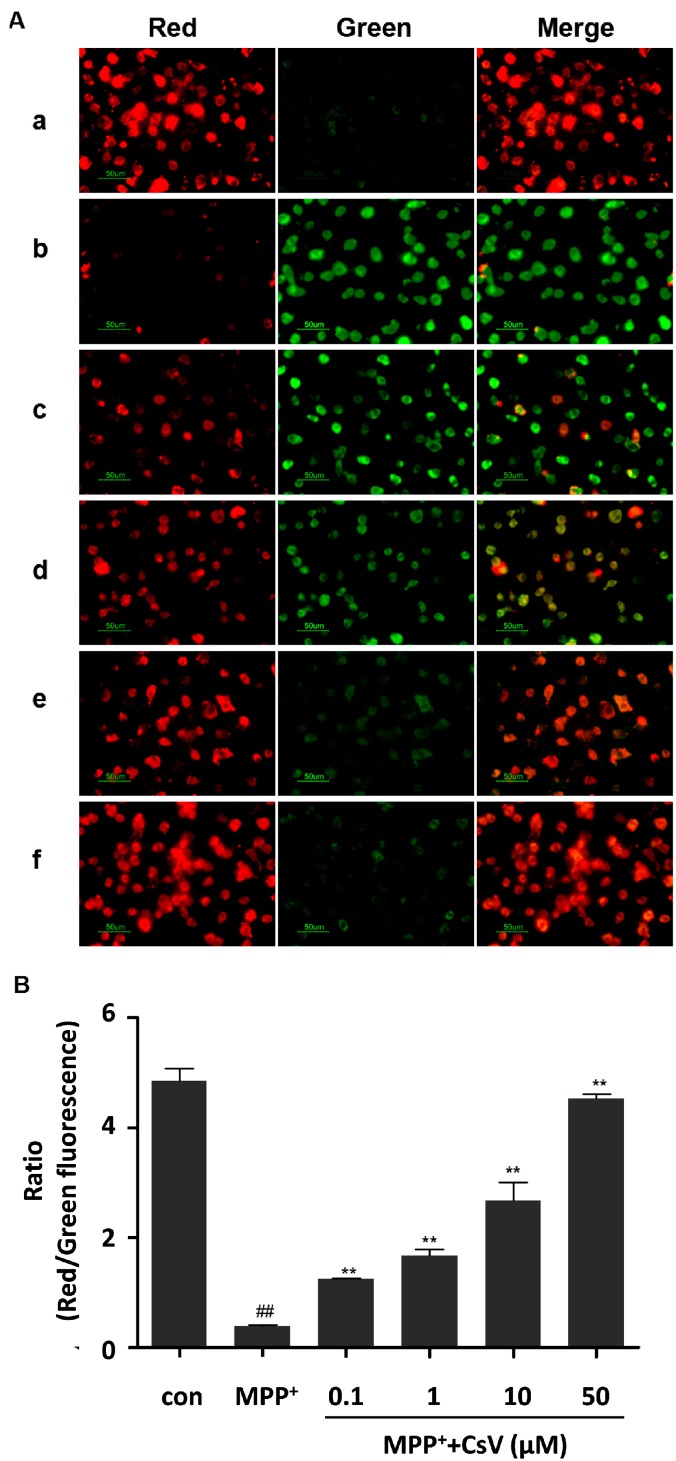
CsV restored MPP^+^-induced mitochondrial membrane potential in SH-SY5Y cells. (**A**) Mitochondrial membrane potential was detected by JC-1 staining. Images are shown as representative from five independent experiments at 400× magnification. Scale bar: 50 µm. (**a**) control (cells without MPP^+^ and CsV); (**b**) 1 mM MPP^+^ treatment alone; (**c**) 0.1 µM CsV with 1 mM MPP^+^; (**d**) 1 µM CsV with 1 mM MPP^+^; (**e**) 10 µM CsV with 1 mM MPP^+^; and (**f**) 50 µM CsV with 1 mM MPP^+^; and (**B**) Ratio of red/green fluorescence. ^##^ *p* < 0.01 *vs.* control group, ** *p* < 0.01 *vs.* MPP^+^ treated alone. Data are expressed as means ± SD (*n* = 5).

JC-1, a lipophilic cationic dye, was used to detect mitochondrial transmembrane potential changes. As shown in Figure 3, control cells exhibited red fluorescence. However, when cells were exposed to MPP^+^ for 24 h, mitochondrial membrane rapidly changed, as shown by increase in green fluorescence and concomitant disappearance of red fluorescence, whereas, different concentrations of CsV treatment (0.1, 1, 10 and 50 µM) for 24 h decreased green fluorescence and increased red fluorescence intensity.

### 2.4. CsV Up-Regulated Sirt1 Protein and Mn-SOD mRNA Expressions in MPP^+^-Treated SH-SY5Y Cells

**Figure 4:**
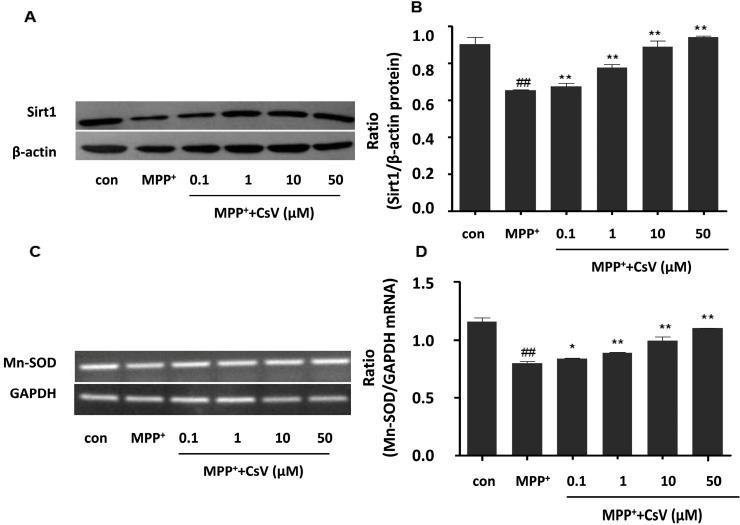
CsV promoted Sirt1 protein and Mn-SOD mRNA expressions in MPP^+^-treated SH-SY5Y cells. (**A**) Western blot analysis of Sirt1 and β-actin protein levels; (**B**) Data quantification of panel (**A**). Western blot was quantified and expressed as the ratio of Sirt1 and β-actin intensity; (**C**) Polymerase chain reaction (PCR) analysis of Mn-SOD and glyceraldehyde-3-phosphate dehydrogenase (GAPDH) mRNA levels; and (**D**) Data quantification of panel (**C**). PCR was quantified and expressed as the ratio of Mn-SOD and GAPDH intensity. ^##^ *p* < 0.01 *vs.* control group, * *p* < 0.05, ** *p* < 0.01 *vs.* MPP^+^ treated alone. Data are expressed as means ± SD (*n* = 3).

To explore the possible mechanisms of the neuroprotective effect, we investigated the effects of CsV on Sirt1/Mn-SOD pathway in SH-SY5Y cells. SH-SY5Y cells were collected for western blot or polymerase chain reaction (PCR) at the end of treatment. As shown in Figure 4A, SH-SY5Y cells exposed to MPP^+^ alone significantly decreased Sirt1 protein level. However, a remarkable increase of Sirt1 protein level was detected in CsV treated-group. MPP^+^ exposure also significantly decreased Mn-SOD mRNA level, which was reversed by CsV treatment (Figure 4B).

### 2.5. CsV Ameliorated MPP^+^-Induced Endoplasmic Reticulum (ER) Stress in SH-SY5Y Cells

**Figure 5:**
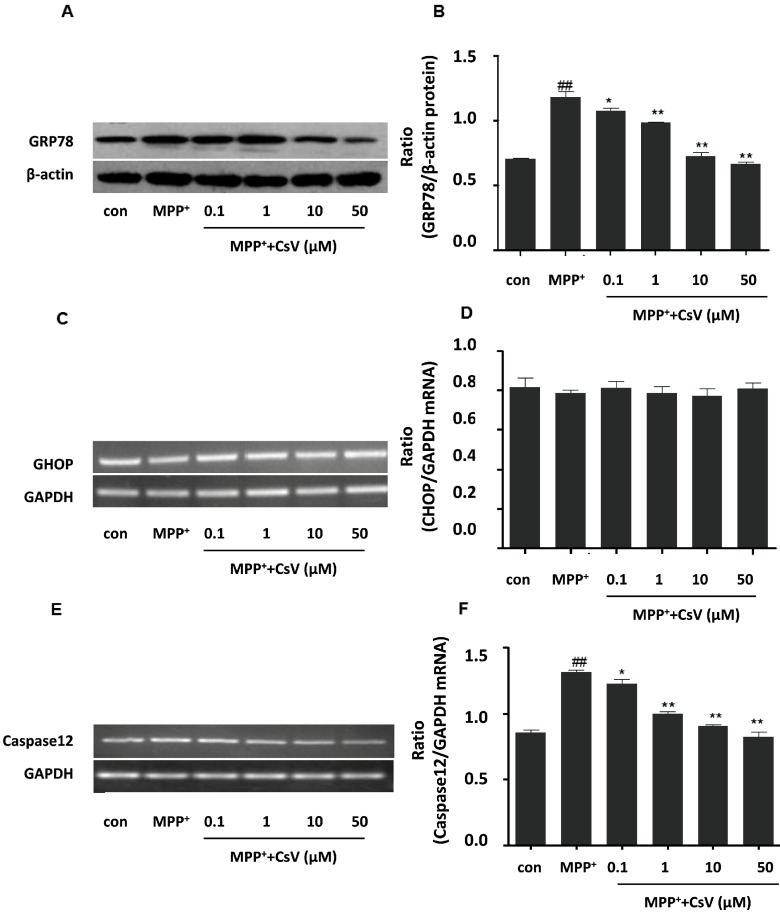
CsV ameliorated MPP^+^-induced endoplasmic reticulum (ER) stress in SH-SY5Y cells. (**A**) Western blot analysis of GRP78 and β-actin protein levels; (**B**) Data quantification of panel (**A**). Western blot was quantified and expressed as the ratio of GRP78 and β-actin intensity; (**C**) PCR analysis of C/EBP homologous protein (CHOP) and GAPDH mRNA levels; (**D**) Data quantification of panel (**C**). PCR was quantified and expressed as the ratio of CHOP and GAPDH intensity; (**E**) PCR analysis of Caspase12 and GAPDH mRNA levels; and (**F**) Data quantification of panel (**E**). PCR was quantified and expressed as the ratio of Caspase12 and GAPDH intensity. ^##^ *p* < 0.01 *vs.* control group, * *p* < 0.05, ** *p* < 0.01 *vs.* MPP^+^ treated alone. Data are expressed as means ± SD (*n* = 3).

To examine the involvement of ER stress in MPP^+^-induced toxicity and CsV protective mechanisms in this study, we examined GRP78 protein, Caspase-12 and CHOP mRNA levels after MPP^+^ and CsV treatment respectively. Our results show that treatment with MPP^+^ lead to increase of GRP78 protein and Caspase-12 mRNA expressions, which are reversed by CsV treatment in a dose-dependent manner. However, there is no significant difference in CHOP mRNA level among these groups. These data suggest that CsV regulate MPP^+^-induced ER stress by repressing GRP78 protein and Caspase-12 mRNA expressions (Figure 5).

### 2.6. CsV Reversed MPP^+^-Induced Apoptosis in SH-SY5Y Cells

**Figure 6:**
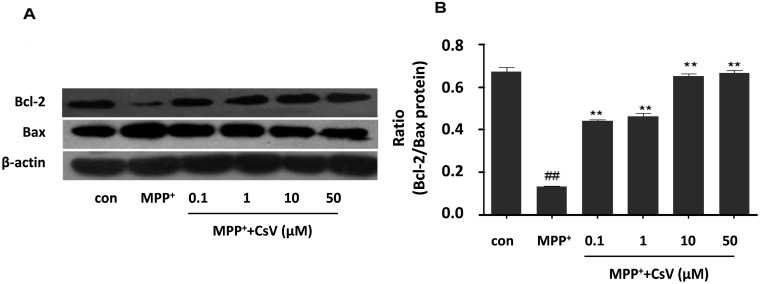
CsV reversed MPP^+^-induced apoptosis in SH-SY5Y cells. (**A**) Western blot analysis of Bcl-2 and Bax; and (**B**) Data quantification of panel (**A**), Western blot was quantified and expressed as the ratio of Bcl-2/Bax intensity. ^##^ *p* < 0.01 *vs.* control group, ** *p* < 0.01 *vs.* MPP^+^ treated alone. Data are expressed as means ± SD (*n* = 3).

Cell survival in the early phase of apoptotic cascade depends mostly on the balance between pro-apoptotic and anti-apoptotic proteins of the Bcl-2 family. Bcl-2 family consists of anti-apoptotic factors such as Bcl-2 and pro-apoptotic factors such as Bax. The ratio of Bcl-2/Bax is a better determinant of cell fate than the absolute concentration of either protein alone [23]. In this study, we tested whether Bcl-2 and Bax protein levels were affected by exposure to MPP^+^ or CsV. The western blot data show that MPP^+^-induced down-regulation of Bcl-2 and up-regulation of Bax are inhibited by CsV treatment (Figure 6).

## 3. Discussion

In the present study, we investigated the neuroprotective effect of CsV in SH-SY5Y cells treated with MPP^+^. Our results show that treatment of SH-SY5Y cells with CsV greatly reduce the MPP^+^-induced neurotoxicity using MTT and LDH assay. Additionally, MPP^+^ exposure significantly elevates ROS and disrupts mitochondrial membrane potential, which are efficiently attenuated by CsV treatment. These results suggest that CsV exhibits strong protective effect against MPP^+^-induced cytotoxicity.

CsV, also called ginsenoside Ro, is one of the main components of SPJ. He *et al.* demonstrated that SPJ acted as a potent antioxidant against acute myocardial ischemia in rats [20]. A number of studies suggested that ROS accumulation was involved in the apoptotic mechanism of MPP^+^-mediated neurotoxicity and might promote the apoptotic processes in PD [24,25]. Other studies have also shown that Astragaloside IV inhibits mitochondrial dysfunction and ROS production, and possesses significant neuroprotective effects in PD model [26]. Consistently, our present studies also demonstrated that CsV exhibited neuroprotective effects in SH-SY5Y cells by depressing mitochondrial dysfunction and ROS production in a dose-dependent manner. The above evidence indicated that the protective effect of CsV against MPP^+^-induced cytotoxicity might be related to reduction of oxidative stress. CsV was able to inhibit ROS production directly by improving the activity of Sirt1 and Mn-SOD. The following studies also corroborate our findings. Chen *et al.* demonstrated that activation of Sirt1 enhanced cellular resistance to oxidative stress [16,27,28]. Sirt1 increased oxidative stress resistance by up-regulating antioxidants such as Mn-SOD [9]. Mn-SOD, located in the mitochondria, contributed to cellular homeostasis against oxidative damage [29]. Mn-SOD converted superoxide to H_2_O_2_ and mitochondrial glutathione peroxidase catalyzes the reduction of H_2_O_2_ to H_2_O [30].

ER, located in eukaryotic cells, is involved in post-translational modifications, folding and synthesis of new membrane and secretary proteins, metabolism and apoptosis [4]. It is important that ER maintains normal cell function and survival in steady state [31]. The neurotoxin MPP^+^ induces oxidative stress damage and mitochondrial dysfunction, causes the accumulation of unfolded or misfolded proteins in ER and results in dysfunction of unfold protein response, leading to ER stress and cell death [18]. Experimental evidence demonstrates that ROS generation and mitochondrial dysfunction leads to ER stress and further accelerates the development of PD [12,32]. ER stress is indicated by increase of GRP78 protein [33], together with its downstream factors, CHOP and Caspase-12, play important roles in ER stress-induced cell apoptosis [34]. We also detected the changes of GRP78 protein, CHOP and Caspase-12 mRNA expression. Our results show that GRP78 protein and Caspase-12 mRNA expression are up-regulated in MPP^+^ group and inhibited by CsV treatment. Therefore, it is concluded that CsV possibly represses MPP^+^-induced ER stress by decreasing the expressions of GRP78 protein and Caspase-12 mRNA.

Both oxidative stress and ER stress lead to cell apoptosis eventually [35]. Bcl-2 family plays a crucial role in cell apoptosis [36]. Previous findings regarding the Bcl-2 proteins indicated that pro-apoptotic family members participate in neuronal death in a variety of PD models [37]. Over-expression of Bcl-2 protects dopaminergic neurons against MPTP-induced neurodegeneration [38]. In the present study, our results show that the decreased of Bcl-2 and increased of Bax in the MPP^+^-treated alone group could be reversed by CsV treatment, which is in agreement with previous studies [39]. Therefore, the effect of CsV on MPP^+^-induced apoptosis might be partially mediated by decreasing the ratio of Bcl-2/Bax.

## 4. Experimental Section

### 4.1. Materials

CsV (purity > 95%) was purchased from Chengdu Purechem-Standard Co., Ltd. (Chengdu, China). Human neuroblastoma SH-SY5Y cells were provided by Huazhong University of Science and Technology (Wuhan, China). DMEM and fetal bovine serum were purchased from Gibco (Grand Island, NY, USA). LDH assay kit was purchased from Nanjing Jiancheng Bioengineering Institute (Nanjing, China). ROS assay kit and JC-1 assay kit were purchased from Beyotime Institute of Biotechnology (Haimen, China). Trizol and PrimeScript^®^ RT reagent Kit were purchased from TaKaRa (Dalian, China). PCR Green Master Mix was purchased from Promega (Madison, WI, USA). PCR primers of CHOP, Caspase-12 and GAPDH were synthesized by Shanghai Shenergy Biocolor BioScience and Technology (Shanghai, China). Monocolonal primary antibodies against GRP78, Bax, Bcl-2 and β-actin were from Santa cruz (Santa Cruz, CA, USA). Sirt1 was from Millipore (Bedford, MA, USA). Other materials were purchased from Sigma-Aldrich (St. Louis, MO, USA).

### 4.2. Cell Culture

SH-SY5Y cells were cultured in DMEM with 10% (*v*/*v*) fetal bovine serum, penicillin (100 U/mL), streptomycin (100 μg/mL) at 37 °C, under 5% CO_2_ in air. The medium was replaced every other day. Cell passaging was done every two days.

### 4.3. MTT Viability Assay

Cell viability was measured by MTT colorimetrically. In brief, cells were seeded into 96-well plates until their adherent. At the indicated time points following MPP^+^ exposure or different concentrations of CsV treatments (0.1, 1, 10 and 50 µM) for 24 h, a 10 μL aliquot of MTT solution was added to the medium to yield a final MTT concentration of 0.5 mg/mL and incubated at 37 °C for 4 h. The MTT solution was removed gently and 200 μL of DMSO was added to each well for 15 min incubation. The absorbance of each sample was measured at 570 nm using a microplate reader (Bio-Rad, Hercules, CA, USA). Net absorbance from the plate of cells cultured with control medium (not treated) was considered to be at 100% cell viability.

### 4.4. Lactate Dehydrogenase (LDH) Release Assay

Logarithmic growth phase of SH-SY5Y cells were seeded in 24-well plates (1 × 10^5^ cells per well). Cells were then randomized to the following groups: control group (normal SH-SYSY cells were cultured under normoxia), MPP^+^ group (the cells were treated with 1 mM MPP^+^ for 24 h), MPP^+^ + CsV group (the cells were co-incubated with 1 mM MPP^+^ and CsV at 0.1, 1, 10 and 50 µM for 24 h). LDH release was measured according to the kit instructions. Absorbance was measured in a microplate reader at a wavelength of 450 nm. Data was expressed as percentage of fluorescence values in control group.

### 4.5. Detection of Intracellular ROS Level

Logarithmic growth phase of SH-SY5Y cells were seeded in 6-well plates (1 × 10^5^ cells per well) on the first day. CsV (0.1, 1, 10 and 50 µM) and MPP^+^ (1 mM) were added to each well for 24 h on the next day. The average level of intracellular ROS was detected in cells loaded with the DCFH-DA probe at 37 °C for 20 min, then washed twice with PBS, The cells were observed and photographed under the fluorescence microscope.

### 4.6. Detection of Mitochondrial Membrane Potential

JC-1 kit was used to measure the mitochondrial membrane potential according to the manufacturer’s instructions after CsV (0.1, 1, 10 and 50 µM) and MPP^+^ (1 mM) were added to each well for 24 h. Briefly, cells cultured in 6-well plates were incubated with JC-1 staining solution for 20 min at 37 °C after treatments and then rinsed twice with JC-1 staining buffer. Finally, cells were observed and photographed under a fluorescence microscope.

### 4.7. Western Blot

Equal amount of protein was electrophoresed and then transferred to PVDF membranes. After blocking the membranes for 60 min in TBST containing 5% non-fat dried milk, membranes were incubated overnight at 4 °C with primary antibodies directed against Sirt1, GRP78, Bcl-2, Bax, and β-actin, followed by HRP-conjugated anti-rabbit or anti-mouse IgG at room temperature for 1 h. Target protein bands were then observed by the enhanced chemiluminescence and tabletted by Kodak film (Eastman Kodak, Rochester, NY, USA). Relative protein levels were quantified by scanning densitometry and analyzed by Image J (Image J Software, Materialize NV, Leuven, Belgium).

### 4.8. Reverse Transcription Polymerase Chain Reaction (RT-PCR)

Total RNA was extracted from SH-SY5Y cells using Trizol reagent. The cDNA was synthesized from total RNA (1 μg) using cDNA synthesis kit according to the supplier’s instructions. The polymerase chain reaction (PCR) was performed using 1 μL cDNA in a total volume of 25 μL reaction. Amplification of all target genes at a concentration of 12.5 μL PCR Green Master Mix. The PCR conditions were presented as follows. After initial denaturation at 94 °C for 3 min, denaturation at 94 °C for 30 s, annealing at 55 °C for 30 s, extension at 72 °C for 30 s, final extension at 72 °C for 7 min, and 30 amplification cycles were carried out. The PCR primers’ sequence are described as follows: CHOP (sense: AACCAGCAGAGGTCACAAGCAC, anti-sense: GCCACTTTCCTTTCATTCTCCTGT), Caspase-12 (sense: AAACATTTGGAGGAGGTCCCA, anti-sense:CTGCTCACACGATTTCCCG), and GAPDH (sense: ACTTCAACAGCGACACCCACTC, anti-sense: TCTCTCTTCCTCTTGTGCTCTTGC). Polymerase chain reaction of GAPDH was chosen as an internal control was performed in the same tubes as for the genes.

### 4.9. Statistical Analysis

Results were represented as means ± SD. All analysis was carried out with GraphPad Prism 5.0 (GraphPad Software, San Diego, CA, USA). Statistical comparisons were determined by one-way ANOVA, followed by two-tailed Student’s *t*-test to determine significant differences between the two groups. *p*-values of less than 0.05 were considered statistically significant.

## 5. Conclusions

In conclusion, CsV attenuates MPP^+^-induced neurotoxicity in SH-SY5Y cells associated with oxidative stress and ER stress. Our results indicate that CsV may be a potential drug to treat PD by down-regulating intracellular ROS, mitochondrial membrane potential, and apoptosis, which are possibly mediated through Sirt1/MnSOD and GRP78/Caspase-12 pathways. However, SH-SY5Y cells cannot fully represent the characteristics of primary cultured neurons, our future work will include primary neurons and *in vivo* models.

## References

[1] Braak H., del Tredici K., Rub U., de Vos R.A., Jansen Steur E.N., Braak E. (2003). Staging of brain pathology related to sporadic Parkinson’s disease. Neurobiol. Aging.

[2] Bloem B.R., Irwin I., Buruma O.J., Haan J., Roos R.A., Tetrud J.W., Langston J.W. (1990). The MPTP model: Versatile contributions to the treatment of idiopathic Parkinson’s disease. J. Neurol. Sci..

[3] Cleeter M.W., Cooper J.M., Schapira A.H. (1992). Irreversible inhibition of mitochondrial complex I by 1-methyl-4-phenylpyridinium: Evidence for free radical involvement. J. Neurochem..

[4] Hauser D.N., Hastings T.G. (2013). Mitochondrial dysfunction and oxidative stress in Parkinson’s disease and monogenic parkinsonism. Neurobiol. Dis..

[5] Moore D.J., West A.B., Dawson V.L., Dawson T.M. (2005). Molecular pathophysiology of Parkinson’s disease. Annu. Rev. Neurosci..

[6] Corbi G., Conti V., Scapagnini G., Filippelli A., Ferrara N. (2012). Role of sirtuins, calorie restriction and physical activity in aging. Front. Biosci..

[7] Wang T., Gu J., Wu P.F., Wang F., Xiong Z., Yang Y.J., Wu W.N., Dong L.D., Chen J.G. (2009). Protection by tetrahydroxystilbene glucoside against cerebral ischemia: Involvement of JNK, Sirt1, and NF-êB pathways and inhibition of intracellular ROS/RNS generation. Free Radic. Biol. Med..

[8] Brunet A., Sweeney L.B., Sturgill J.F., Chua K.F., Greer P.L., Lin Y., Tran H., Ross S.E., Mostoslavsky R., Cohen H.Y. (2004). Stress-dependent regulation of FOXO transcription factors by the Sirt1 deacetylase. Science.

[9] Alcendor R.R., Gao S., Zhai P., Zablocki D., Holle E., Yu X., Tian B., Wagner T., Vatner S.F., Sadoshima J. (2007). Sirt1 regulates aging and resistance to oxidative stress in the heart. Circ. Res..

[10] Yun J.M., Chien A., Jialal I., Devaraj S. (2012). Resveratrol up-regulates Sirt1 and inhibits cellular oxidative stress in the diabetic milieu: Mechanistic insights. J. Nutr. Biochem..

[11] Tang B.L. (2006). Sirt1, neuronal cell survival and the insulin/IGF-1 aging paradox. Neurobiol. Aging.

[12] Yoon H., Kim D.S., Lee G.H., Kim K.W., Kim H.R., Chae H.J. (2011). Apoptosis induced by manganese on neuronal sk-n-mc cell line: endoplasmic reticulum (er) stress and mitochondria dysfunction. Environ. Health Toxicol..

[13] Egawa N., Yamamoto K., Inoue H., Hikawa R., Nishi K., Mori K., Takahashi R. (2011). The endoplasmic reticulum stress sensor, ATF6á, protects against neurotoxin-induced dopaminergic neuronal death. J. Biol. Chem..

[14] Hara H., Kamiya T., Adachi T. (2011). Endoplasmic reticulum stress inducers provide protection against 6-hydroxydopamine-induced cytotoxicity. Neurochem. Int..

[15] Hoozemans J.J., Scheper W. (2012). Endoplasmic reticulum: The unfolded protein response is tangled in neurodegeneration. Int. J. Biochem. Cell Biol..

[16] Chen Z., Peng I.C., Cui X., Li Y.S., Chien S., Shyy J.Y. (2010). Shear stress, Sirt1, and vascular homeostasis. Proc. Natl. Acad. Sci. USA.

[17] Fujita E., Kouroku Y., Jimbo A., Isoai A., Maruyama K., Momoi T. (2002). Caspase-12 processing and fragment translocation into nuclei of tunicamycin-treated cells. Cell Death Differ..

[18] Choi A.Y., Choi J.H., Yoon H., Hwang K.Y., Noh M.H., Choe W., Yoon K.S., Ha J., Yeo E.J., Kang I. (2011). Luteolin induces apoptosis through endoplasmic reticulum stress and mitochondrial dysfunction in Neuro-2a mouse neuroblastoma cells. Eur. J. Pharmacol..

[19] Han L.K., Zheng Y.N., Yoshikawa M., Okuda H., Kimura Y. (2005). Anti-obesity effects of chikusetsusaponins isolated from *Panax japonicus* rhizomes. BMC Complement. Altern. Med..

[20] He H., Xu J., Xu Y., Zhang C., Wang H., He Y., Wang T., Yuan D. (2012). Cardioprotective effects of saponins from *Panax japonicus* on acute myocardial ischemia against oxidative stress-triggered damage and cardiac cell death in rats. J. Ethnopharmacol..

[21] Li Y.G., Ji D.F., Zhong S., Shi L.G., Hu G.Y., Chen S. (2010). Saponins from *Panax japonicus* protect against alcohol-induced hepatic injury in mice by up-regulating the expression of GPX3, SOD1 and SOD3. Alcohol Alcohol..

[22] Hosono-Nishiyama K., Matsumoto T., Kiyohara H., Nishizawa A., Atsumi T., Yamada H. (2006). Suppression of Fas-mediated apoptosis of keratinocyte cells by chikusetsusaponins isolated from the roots of *Panax japonicus*. Planta Med..

[23] Li C., Zhang J., Li J., Li H., Lin Q., Li B. (2012). Research on chemical constituents and biological activities of rhizoma of *Panax japonicus*. Guid. J. Tradit. Chin. Med. Pharm..

[24] Zhang Z.G., Wu L., Wang J.L., Yang J.D., Zhang J., Zhang J., Li L.H., Xia Y., Yao L.B., Qin H.Z. (2012). Astragaloside IV prevents MPP^+^-induced SH-SY5Y cell death via the inhibition of Bax-mediated pathways and ROS production. Mol. Cell. Biochem..

[25] Kim I.S., Choi D.K., Jung H.J. (2011). Neuroprotective effects of vanillyl alcohol in *Gastrodia elata* Blume through suppression of oxidative stress and anti-apoptotic activity in toxin-induced dopaminergic MN9D cells. Molecules.

[26] Wang Y.H., Yu H.T., Pu X.P., Du G.H. (2013). Baicalein prevents 6-hydroxydopamine-induced mitochondrial dysfunction in SH-SY5Y cells via inhibition of mitochondrial oxidation and up-regulation of DJ-1 protein expression. Molecules.

[27] Yan W., Fang Z., Yang Q., Dong H., Lu Y., Lei C., Xiong L. (2013). Sirt1 mediates hyperbaric oxygen preconditioning-induced ischemic tolerance in rat brain. J. Cereb. Blood Flow Metab..

[28] He W., Wang Y., Zhang M.Z., You L., Davis L.S., Fan H., Yang H.C., Fogo A.B., Zent R., Harris R.C. (2010). Sirt1 activation protects the mouse renal medulla from oxidative injury. J. Clin. Investig..

[29] Xin Y.F., Wan L.L., Peng J.L., Guo C. (2011). Alleviation of the acute doxorubicin-induced cardiotoxicity by *Lycium barbarum* polysaccharides through the suppression of oxidative stress. Food Chem. Toxicol..

[30] Li S., Yan T., Yang J.Q., Oberley T.D., Oberley L.W. (2000). The role of cellular glutathione peroxidase redox regulation in the suppression of tumor cell growth by manganese superoxide dismutase. Cancer Res..

[31] Chen J.C., Wu M.L., Huang K.C., Lin W.W. (2008). HMG-CoA reductase inhibitors activate the unfolded protein response and induce cytoprotective GRP78 expression. Cardiovasc. Res..

[32] Li X., Chen W., Zhang L., Liu W.B., Fei Z. (2013). Inhibition of store-operated calcium entry attenuates MPP^+^-induced oxidative stress via preservation of mitochondrial function in PC12 cells: Involvement of Homer1a. PLoS One.

[33] Wang Z.C., Wang J.F., Li Y.B., Guo C.X., Liu Y., Fang F., Gong S.L. (2013). Involvement of endoplasmic reticulum stress in apoptosis of testicular cells induced by low-dose radiation. J. Huazhong Univ. Sci. Technol. Med. Sci..

[34] Lakshmanan A.P., Thandavarayan R.A., Palaniyandi S.S., Sari F.R., Meilei H., Giridharan V.V., Soetikno V., Suzuki K., Kodama M., Watanabe K. (2011). Modulation of AT-1R/CHOP-JNK-Caspase12 pathway by olmesartan treatment attenuates ER stress-induced renal apoptosis in streptozotocin-induced diabetic mice. Eur. J. Pharm. Sci..

[35] Jin M.L., Park S.Y., Kim Y.H., Oh J.I., Lee S.J., Park G. (2014). The neuroprotective effects of cordycepin inhibit glutamate-induced oxidative and ER stress-associated apoptosis in hippocampal HT22 cells. Neurotoxicology.

[36] Henshall D.C., Engel T. (2013). Contribution of apoptosis-associated signaling pathways to epileptogenesis: Lessons from Bcl-2 family knockouts. Front. Cell. Neurosci..

[37] Levy O.A., Malagelada C., Greene L.A. (2009). Cell death pathways in Parkinson’s disease: Proximal triggers, distal effectors, and final steps. Apoptosis.

[38] Offen D., Beart P.M., Cheung N.S., Pascoe C.J., Hochman A., Gorodin S., Melamed E., Bernard R., Bernard O. (1998). Transgenic mice expressing human Bcl-2 in their neurons are resistant to 6-hydroxydopamine and 1-methyl-4-phenyl-1,2,3,6-tetrahydropyridine neurotoxicity. Proc. Natl. Acad. Sci. USA.

[39] Blum D., Torch S., Lambeng N., Nissou M., Benabid A.L., Sadoul R., Verna J.M. (2001). Molecular pathways involved in the neurotoxicity of 6-OHDA, dopamine and MPTP: Contribution to the apoptotic theory in Parkinson’s disease. Prog. Neurobiol..

